# Communities of Arbuscular Mycorrhizal Fungi and Their Effects on Plant Biomass Allocation Patterns in Degraded Karst Grasslands of Southwest China

**DOI:** 10.3390/jof11070525

**Published:** 2025-07-16

**Authors:** Wangjun Li, Xiaolong Bai, Dongpeng Lv, Yurong Yang

**Affiliations:** 1 College of Ecological Engineering, Guizhou University of Engineering Science, Bijie 551700, China; teesn470@gues.edu.cn (W.L.); baixiaolong@gues.edu.cn (X.B.); 2 Key Laboratory of Ecological Microbial Remediation Technology of Yunnan Higher Education Institutes, Dali University, Dali 671003, China; 911882@dali.edu.cn; 3 State Environmental Protection Key Laboratory of Wetland Ecology and Vegetation Restoration, School of Environment, Northeast Normal University, Changchun 130117, China

**Keywords:** arbuscular mycorrhizal fungi, plant biomass allocation, subalpine grassland, *Festuca ovina*, rocky desertification

## Abstract

The biomass allocation patterns between aboveground and belowground are an essential functional trait for plant survival under a changing environment. The effects of arbuscular mycorrhizal fungi (AMF) communities on plant biomass allocation, particularly in degraded *Festuca ovina* grasslands in ecologically fragile karst areas, remain unclear. Therefore, we conducted a field investigation combined with a greenhouse experiment to explore the importance of AMF compared to bacteria and fungi for plant biomass allocation. The results showed that plant biomass in degraded grasslands exhibited allometric biomass allocation, contrasting with isometric partitioning in non-degraded grasslands. AMF, not bacteria or fungi, were the primary microbial mediators of grassland degradation effects on plant biomass allocation based on structural equation modeling. The greenhouse experiment demonstrated that the selected AMF keystone species from the field study performed according to ecological network analysis, particularly multi-species combinations, enhanced the belowground biomass allocation of *F. ovina* under rocky desertification stress compared to single-species inoculations, through decreasing soil pH, enhancing alkaline phosphatase (ALP) activity, and increasing the expression level of AMF-inducible phosphate transporter (*PT4*). This study highlights the critical role of the AMF community, rather than individual species, in mediating plant survival strategies under rocky desertification stress.

## 1. Introduction

Plant biomass allocation is a crucial plant trait for understanding plant survival strategies and growth optimization and predicting terrestrial carbon storage and ecosystem functioning [1,2]. Moreover, it can reflect environmental adaptation strategies, which are commonly viewed as an interactive result between abiotic and biotic variables [3,4]. Under a changing environment, plants evolve complex physiological and biochemical adaptations to survive and succeed in stressed habitats [5]. Simultaneously, plants also develop cost-effective strategies by forming mutual relationships with symbiotic microorganisms to cope with threats to plant growth and development, especially in extremely degraded habitats [6]. Nevertheless, microorganisms are very diverse in different ecosystems, including Archaea, Bacteria, and Eukarya. These microbes can form complex co-associations with plants and are vital to plant growth and stress tolerance. However, the relative contributions of soil microbes in shaping plant adaptive strategies, such as biomass allocation patterns in response to changing environments, remain unclear.

Two important theories have been proposed and are commonly used to assess plant biomass allocation patterns [7]. An isometric partitioning theory or allometric partitioning theory suggests that plant aboveground biomass (AGB) scales nearly isometrically or allometrically with plant belowground biomass (BGB) and that the scaling slope remains unaffected by various environmental factors [8]. The isometric partitioning theory has been extensively studied at both the individual and community levels, and a previous study found that AGB rose isometrically with an increase in BGB, with constant scaling exponents of 1.06 (95% CI, 1.05–1.08) and 1.02 across 257 plant species and 6 grassland types, respectively [9,10]. The APT for plant biomass allocation organs is derived from a strictly analytical approach according to the metabolic theory of ecology, which is regarded as the result of allometric constraints of evolution [11]. Yet, the isometric partitioning theory and allometric partitioning theory remain controversial among different plant species because they only regard plant biomass allocation patterns as size-independent and do not reveal how environmental variables affect biomass allocation [12].

Known as another prevalent theory for plant biomass allocation, optimal partitioning theory proposes that plants in resource-limited habitats prefer to allocate biomass to obtain the limited resource [8]. The optimal partitioning theory suggests that plants would allocate a greater proportion of biomass to BGB to promote soil nutrient and water uptake when they are suffering from soil salinity stress. By contrast, plants tend to allocate more energy to AGB to maximize growth by accessing more light [12]. Although the isometric partitioning theory indicates that AGB scales isometrically with BGB based on plant size [13], it seems that plant biomass allocation is closely related to biotic and abiotic factors, as predicted by optimal partitioning theory when the environmental stress gradient is taken into account [14]. Nevertheless, whether plant biomass allocation follows the same patterns at both the individual and ecosystem levels along a rocky desertification stress gradient is still uncertain.

Global warming, air pollution, and land degradation associated with a rapid increase in human population continue to be major environmental issues. Among them, soil degradation, which may cause a reduction in plant biomass and growth [15], is becoming an important ecological indicator in many parts of the world. Several plants can develop distinctive strategies when exposed to harsh conditions, as reflected in morphological plasticity and biomass re-allocation [16]. However, above- and belowground plant organs have different physiological characteristics, and their responses to the same environmental factor are probably inconsistent. For instance, ecosystem degradation increased plant BGB while reducing plant AGB in an alpine grassland [17]. To date, most studies focusing on biomass partitioning tend to emphasize AGB more than BGB [18], whereas research on plant biomass allocation patterns at the community and individual levels in rocky desertification degraded grasslands remains scarce.

Some plants can also develop a cost-effective strategy in response to abiotic stress by forming symbiotic mutualism with microbes [19]. Arbuscular mycorrhizal fungi (AMF) exist widely in soils and establish mutualistic associations with most terrestrial plants [6]. Previous research has demonstrated that AMF play a crucial role in plant growth and stress tolerance by promoting soil nutrient uptake and regulating plant nutrient stoichiometry [20]. Relief of host nutrient limitation as a consequence of AMF inoculation can be expected to increase AGB. Unfortunately, there is little systematic study on how plant biomass trade-offs between ABG and BGB are affected by AMF and their influencing pathways, which improve plant adaptability.

*Festuca ovina* grasslands (climax community of subalpine zones) in ecologically fragile karst areas of southwest China are essential for biodiversity protection, ecosystem service maintenance, and animal husbandry development. Soil rocky desertification and long-term overgrazing are mostly responsible for the degradation of *F*. *ovina* grasslands. The subalpine zone in karst regions is largely covered by *F*. *ovina*, which grows well in rocky desertification soils and has gradually become the dominant species and a promising candidate for the restoration of degraded grassland [21]. When facing rocky desertification stress, the roots of *F*. *ovina* were widely colonized by AMF, which enhanced the growth and development of plants [22]. The contribution of AMF symbiosis to the adaptive strategies of grasses to grassland degradation is not well-documented, particularly regarding plant biomass allocation patterns. Therefore, we investigated the plant biomass allocation patterns in *F*. *ovina* grassland under different degrees of rocky desertification to answer the following three main questions: (i) are AMF more important to plant biomass allocation than other microorganisms (e.g., bacteria and fungi)? (ii) what are the keystone species of AMF that affect plant biomass allocation? and (iii) what are the mechanisms by which AMF mediate plant biomass allocation under rocky desertification stress?

## 2. Materials and Methods

### 2.1. Experiment 1: Field Investigation

#### 2.1.1. Study Sites and Sampling

This study was conducted in subalpine *Festuca ovina* grasslands within Bijie City (26°45′06″–27°09′21″ N, 104°05′54″–104°47′21″ E), Guizhou province, China (Figure S1). Situated at around 1500 m elevation, the study area experiences a subtropical humid monsoon climate. The area has a mean annual temperature of 10–15 °C, and the average annual temperature for 50 years is 14.7 °C, with January being the coldest month (4.6 °C) and July being the warmest (23.7 °C). The area receives a mean annual precipitation between 849 and 1399 mm, and the seasonal distribution peaks in summer (~45.7% of annual total), with July being the wettest month, while winter receives minimal rainfall (~5.0% of annual total). The annual sunshine duration ranges between 1096 and 1769 h, and the frost-free season spans from 245 to 290 days in the area. According to the FAO classification, the soil in the region belongs to the Calcisols profile, exhibiting a loam texture with the following particle size distribution: clay (<0.001 mm): 17.69%; silt (0.001–0.05 mm): 42.84%; and sand (0.05–1.00 mm): 39.47%. High levels of calcium in the soil parent material, coupled with a thin soil layer and high evaporation rates, are the primary causes leading to soil rocky desertification and subsequent grassland degradation. Vegetation in this region is predominantly composed of *Festuca ovina* (with a biomass accounting for >90% of the community biomass), which is the climax community in this area. Research has demonstrated that AMF are extensively distributed in the soils of this area [23]. These fungi form symbiotic relationships with plant roots, even in heavily degraded grasslands, playing a crucial role in plant survival and growth under harsh environments, indicating the significant importance of AMF to enhance plant resilience in degraded ecosystems [24]. In summary, the *Festuca ovina* grassland in this region exemplifies an ecosystem facing significant challenges due to harsh climatic and edaphic conditions. However, the resilience of this ecosystem, supported by symbiotic relationships between plants and AMF, offers valuable insights into strategies for the ecological restoration and sustainable management of degraded grasslands.

In our investigation of grassland degradation (Table S1), we identified the following five distinct *Festuca ovina* grasslands based on the degree of rocky desertification degradation: non-degraded grassland (NDG), lightly degraded grassland (LDG), moderately degraded grassland (MDG), heavily degraded grassland (HDG), and severely degraded grassland (SDG), according to the industry standard of the State Forestry Administration (LY/T 1840–2009) [25]. This classification was derived from metrics such as vegetation coverage, aboveground plant biomass, soil pH, and electrical conductivity (EC). At each site with the same degree of *Festuca ovina* grassland degradation, five plots (20 m × 20 m) within walking distance were established to minimize geographical differences such as temperature and precipitation. Within each plot, five replicate quadrats (50 cm × 50 cm) were placed along a transect line at 6 m intervals. In each quadrat, vegetation height was measured by recording the vertical height of the tallest plants. In each quadrat, two soil cores (8 cm diameter) of 20 cm depth were collected with a soil auger for soil and root sampling. The soil coating the plant roots was collected and then mixed thoroughly in tightly sealed zip-lock bags to make one composite sample within each plot. All roots were washed, dried, and then weighed to assess plant BGB. The aboveground biomass (AGB) of plants was clipped with scissors for biomass assessment. A total of 125 composite soil samples (5 sites × 5 plots × 5 quadrats) were collected and conveyed to the laboratory in cooled boxes. After the thorough removal of surface organic materials and fine roots, one fraction of the sample was stored at 4 °C for the analysis of soil properties, while another fraction was sieved and stored at −80 °C for DNA extraction.

#### 2.1.2. Plant Biomass and Soil Properties

To remove adhering soil particles, biomass samples from each quadrat were carefully washed in a distilled water bath. Subsequently, the aboveground and belowground biomass parts were dried in an oven at 60 °C for 72 h and weighed using a digital balance for AGB and BGB determination. The stored soil samples (4 °C) were then air-dried, ground, and passed through a sieve (2 mm) for further analysis. The Walkley–Black method was employed to assess the soil organic carbon (SOC) content [26]. Using the Kjeldahl method, the total nitrogen (TN) content was determined [27], while the alkaline hydrolysis diffusion method was used to evaluate the available nitrogen (AN) content [28]. The total phosphorus (TP) content was assayed based on digestion with a mixture of HF and HClO_4_ and was then determined using spectrophotometry [29], whereas Olsen’s method was employed to assess the available phosphorus (AP) content [30]. A 1:5 soil–water extract was employed to measure soil pH and EC, which were then assessed using the METTLER Toledo FE28 pH meter (Mettler-Toledo Instruments Co., Ltd., Shanghai, China) and Leici DDB−303A conductivity meter (Leici Instrument Co., Ltd., Shanghai, China) [31], respectively. The concentrations of exchangeable cations (Na^+^, K^+^, Ca^2+^, and Mg^2+^) in the soil samples (1:5 soil–water suspension) were analyzed with the Model 410 Flame Photometer (Sherwood Scientific Co., Ltd., Cambridge, UK). We employed the neutralization method to quantify the concentrations of carbonate (CO_3_^2−^) and bicarbonate (HCO_3_^−^) in the soil, while we used a DX−300 ion chromatography system (Dionex, Sunnyvale, CA, USA) to analyze the chloride (Cl^−^) and sulfate (SO_4_^2−^) concentrations [32]. The total C concentration was determined using the titration method [33]. To assess the potential for sodium-induced soil dispersion, we calculated the following two key metrics: sodium adsorption ratio (SAR) and exchangeable sodium percentage (ESP). The calculation methods employed in this study were based on established procedures outlined in our previous research [34,35].

#### 2.1.3. DNA Extraction, PCR Amplification, and Illumina Sequencing

Detailed information about the DNA extraction, PCR amplification, Illumina sequencing, and the process of sequencing data is shown in the Supplementary Materials. Briefly, the total DNA was extracted using ~0.5 g of frozen soil sample according to the DNA extraction kit manufacturer’s instructions (OMEGA, Biotek, Norcross, GA, USA). After confirming the yield and purity of the DNA extracted through 1.5% agarose gel electrophoresis and a NanoDrop 2000 nucleic acid quantifier (Thermo Scientific Inc., Waltham, MA, USA), the bacteria 16S rRNA gene V3−V4 hypervariable region and fungal ITS genes were amplified using the 338F/806R and ITS1/ITS2 universal primers [36,37,38,39], respectively. Nested PCR was performed with primer sets of AML1/AML2 and AMV4.5NF/AMDGR to amplify specific regions of AMF ribosomal RNA genes [40,41]. The first PCR round employed a primer pair (AML1/AML2) specific to Glomeromycota, while a more specific primer set (AMV4.5NF/AMDGR) was utilized to further amplify a smaller target region (~300 bp) [42]. The raw reads were demultiplexed and quality filtered using fastp (v0.20.0). Paired-end reads were then merged using FLASH (v1.2.7). Operational Taxonomic Units (OTUs) were clustered from the processed sequences at a 97% similarity threshold using UPARSE (v7.1), with chimeric sequences removed by Uchime. Taxonomy was assigned using the SILVA (v119), UNITE (v7.0), and MaarjAM databases (https://maarjam.ut.ee/, accessed on 8 January 2021, status June 2019, 384 virtual taxa (VT)) with a 70% confidence threshold for bacterial, fungal, and AMF communities, respectively. Finally, sequence depth normalization was performed across all soil samples before further analysis. The microbial diversity at different study sites was reflected by the indices of observed species (Sobs), Chao1, and Shannon, which were calculated in R software (version 3.6.2) using the vegan package (version 2.5–2) [43].

### 2.2. Experiment 2: Greenhouse Experiment

#### 2.2.1. Growth Substrate, PLANT Seeds, and Fungal Inoculum

Soil samples and plant seeds were all collected from the same study sites as previously described. Four AMF strains, *Glomus mosseae* (GLM, now *Funneliformis mosseae*), *Glomus intraradices* (GLI, now *Rhizophagus intraradices*), *Acaulospora laevis* (ACL), and *Diversispora spurca* (DIS), were isolated onsite [44]. The selection of these four AMF strains was based on their ecological specialization and prevalence in the field study. Utilizing a powerful statistical technique called ecological network analysis (ENA), we identified these AMF genera as being particularly characteristic of specific habitats and exhibiting a relatively high abundance within the study area.

#### 2.2.2. Experimental Design

To examine the effects of AMF and rocky desertification stress on *F*. *ovina*, a dominant grass species in subalpine grasslands, we employed a comprehensive experimental design. The experiment was conducted in the greenhouse of Guizhou University of Engineering Science, Bijie, China, for 95 days. The temperature ranged from 20 to 35 °C, and the relative air humidity ranged from 55 to 87%. The experiment consisted of a randomized complete block design with the following six inoculation treatments: (i) control plants not inoculated with AMF (NM), (ii) plants inoculated with the AMF *G. mosseae* strain (GLM), (iii) plants inoculated with the AMF *G*. *intraradices* strain (GLI), (iv) plants inoculated with the AMF *A*. *laevis* strain (ACL), (v) plants inoculated with the AMF *D*. *spurca* strain (DIS), and (vi) plants inoculated with a mixture of the four AMF strains (MIX). The following five degrees of grassland rocky desertification were created through regulating the variation in soil-to-gravel ratio [45]: RD1, 100% soil; RD2, a mixture of 85% soil and 15% gravel; RD3, a mixture of 70% soil and 30% gravel; RD4, a mixture of 55% soil and 45% gravel; and RD5, a mixture of 40% soil and 60% gravel. Each treatment had five replicates for a total of 150 plastic pots (Supplementary Materials).

#### 2.2.3. Laboratory Analysis

Following 80 days of exposure to rocky desertification stress (total growth period: 95 days), the plants were harvested for further analysis. After growth, we carefully collected all the plants from each pot. We separated the tops (shoots) from the roots, then dried them in an oven (60 °C) for three days until they stopped losing weight. Finally, we weighed each dried plant sample for plant biomass on a digital scale. We assessed the responses of plants to the interactive effects of mycorrhizal inoculation and rocky desertification stress by measuring the concentrations of C, N, and P in both AGB and BGB. Established methods were employed for these analyses, including the Walkley−Black method for C [26], the Kjeldahl method for N [27], and the Molybdenum Blue method for P [29]. The P−Olsen method was employed to measure the concentration of available phosphorus (AP) [30]. Additionally, we investigated potential changes in plant gene expression related to phosphorus acquisition. We focused on the *PT4* gene, acknowledged for its role in phosphate transport. The gene sequence was retrieved from the Transcriptome Database of *Leymus chinensis* [46]. To measure the *PT4* transcript levels in root samples, quantitative real-time PCR (qRT−PCR) was employed. To ensure data accuracy, the qRT−PCR assays were conducted with three biological replicates (separate RNA extractions) and three technical replicates per treatment (Table S2). Using the 2^−ΔΔCT^ method, the relative gene expression levels were calculated [47].

### 2.3. Statistical Analysis

To ensure the validity of our subsequent analyses, we first used the Kolmogorov–Smirnov test to assess the normal distribution of raw data and the residuals. Levene’s test was then employed to confirm equal variances between groups. Following these initial checks, three-way analysis of variance (ANOVA) was employed to detect notable distinctions in plant biomass, nutrient concentrations, and allocation patterns (aboveground vs. belowground biomass) attributable to rocky desertification stress, mycorrhizal inoculation treatments, and the diversity of AMF. Duncan’s test (*p* < 0.05) was used as a post hoc analysis to pinpoint specific treatment groups that differed significantly from each other. It should be noted that all significance levels were set at a stringent 5% (two-tailed test). To obtain a more comprehensive insight into the complex interactions between the biotic and abiotic factors influencing plant biomass allocation, we utilized structural equation modeling (SEM) implemented with AMOS software (version 24). Additionally, we explored the correlation between ABG and BGB using standardized major axis (SMA) regression implemented in R software (version 3.6.2). To characterize allometric relationships between plant organs, we logarithmically transformed both ABG and BGB data. Subsequently, SMA regression with R software (version 3.6.2) and the SMATR package was used to determine allometric slopes and intercepts [48]. These values allowed us to assess whether the relationship between ABG and BGB was isometric (scaling slope close to 1.0). We further investigated the optimal partitioning theory along environmental gradients. Size-corrected biomass ratios (AGB*/BGB*) were calculated based on residuals from the allometric regressions between total plant biomass and the biomass of each organ (AGB and BGB) [49]. To identify the key factors influencing plant biomass allocation patterns, we employed random forest modeling using the randomForest package (version 4.7−1) in R [50]. Finally, we investigated potential co-occurrence patterns within the AMF communities. A correlation matrix based on Spearman’s rank correlation coefficient was constructed to identify significantly correlated AMF Operational Taxonomic Units (OTUs) (*p* < 0.05, *r* > 0.6). These significantly correlated OTUs were then used to build a co-occurrence network visualized with Gephi software (version 0.9.7) [51]. In a network topology analysis, each node’s functional role was determined using within-module connectivity (Zi) and among-module connectivity (Pi) to categorize potential keystone species (network hubs, module hubs, and connectors) which have significant contributions to the stability and resistance of the microbial network structure and functioning [52]. In this study, the species in the networks were classified into four subclasses: peripheral nodes (Zi ≤ 2.5 and 0 ≤ Pi ≤ 0.62), connectors (Zi ≤ 2.5 and Pi > 0.62), module hubs (Zi > 2.5 and Pi ≤ 0.62), and network hubs (Zi > 2.5 and Pi > 0.62).

## 3. Results

### 3.1. Soil Properties in Grasslands with Different Degrees of Degradation

Soil pH (ranging from 6.39 to 7.97) increased significantly with an increasing grassland degradation degree (*p* < 0.05, Table 1). Soil EC in SDG (475 μS m^−1^) and HDG (439 μS m^−1^) was larger compared to NDG, while there was no difference in CEC among study sites (*p* > 0.05). ESP and SAR in HDG were larger than those in NDG, although no difference in SAR was found between MDG and HDG.

Soil SOC, TP, AP, and AN concentrations decreased significantly with an increasing degradation degree, but TN did not vary among NDG, LDG, and MDG. SOC, TP, AP, and AN concentrations decreased by 23.53%, 8.70%, 35.6%, and 13.7% in SDG, respectively, compared to NDG. However, no significant differences in soil SOC, TP, AP, and AN were detected between NDG and LDG (*p* > 0.05).

### 3.2. Microbial Communities in Grasslands with Different Degrees of Degradation

**Figure 1:**
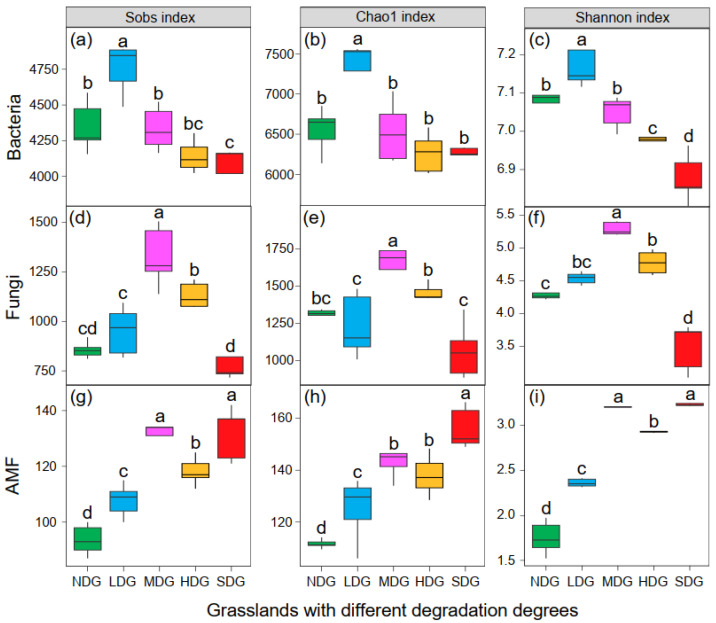
Richness (Sobs index and Chao1 index) and diversity (Shannon index) indices of the bacterial (**a**–**c**), fungal (**d**–**f**), and AMF (**g**–**i**) communities in *Festuca ovina* grasslands with different degrees of rocky desertification. The values (*n* = 5) followed by the same letter do not differ significantly at *p* < 0.05 according to Duncan’s multiple range test. NDG, non-degraded grassland; LDG, lightly degraded grassland; MDG, moderately degraded grassland; HDG, heavily degraded grassland; SDG, severely degraded grassland.

In the current study, 2,634,459, 2,151,298, and 1,407,578 high-quality reads were assessed for bacteria, fungi, and AMF, respectively. The sequencing process captured a significant portion of the microbial communities in the samples, as indicated by the high coverage values (above 0.99). This allowed us to identify a diverse range of bacteria (14,698 OTUs), fungi (6744 OTUs), and AMF (2752 OTUs) based on their genetic similarity (97% threshold). As shown in Figure 1, strong differences in bacterial, fungal, and AMF richness and diversity were found among study sites, as revealed by the Sobs, Chao1, and Shannon indices. Both bacterial and fungal diversity increased first and then decreased with an increasing degradation degree, while AMF diversity increased. The highest bacterial and fungal richness (Sobs and Chao1) and diversity (Shannon indexes) were found in LDG and MDG, respectively. By contrast, the highest AMF richness (Chao1 index, 156.14) and diversity (Shannon index, 3.23) were both detected at the SDG site. However, no differences in the Sobs and Shannon indices were found between MDG and SDG.

### 3.3. Relationship Between Microbial Community and Plant Biomass Allocation Patterns

**Figure 2:**
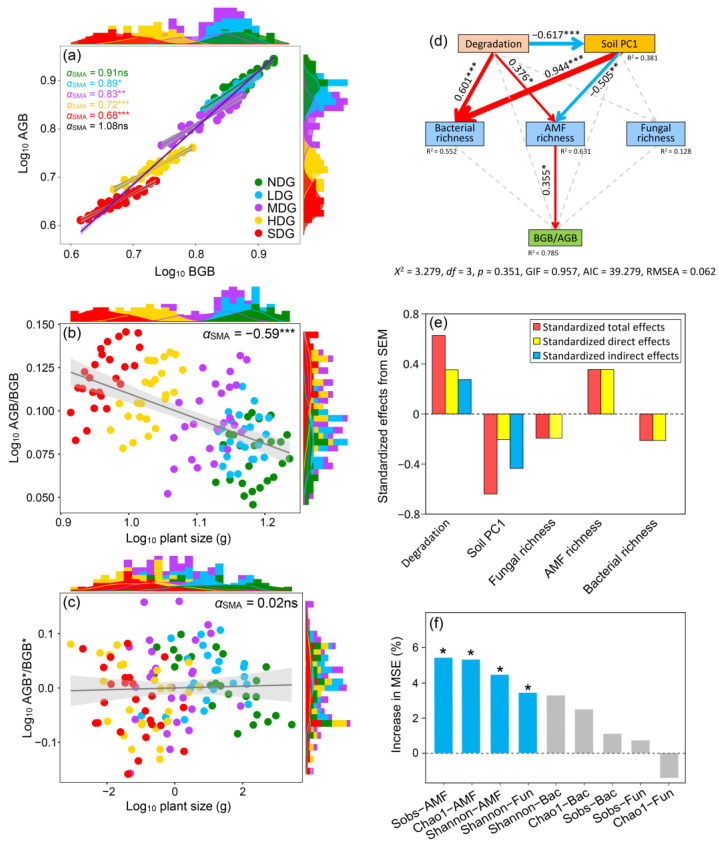
Standardized major axis (SMA) regressions between shoot and root biomasses of *Festuca ovina* grown in grasslands with different degrees of rocky desertification. (**a**–**c**) General allometric relationships of shoot biomass (AGB) versus root biomass (BGB). The solid line and shaded areas show the best fit and 95% CI of the fit test, respectively. (**d**,**e**) Structural equation model (SEM) of the effects of grassland degradation, soil properties, and soil microbial richness (Chao1 index) on plant biomass allocation patterns. (**f**) Random forest model showing mean predictor importance of microbial richness on plant biomass allocation patterns. ns, not significant; * *p*  <  0.05; ** *p*  <  0.01; and *** *p*  <  0.001.

The biomass allocation between AGB and BGB supported an allometric relationship across a gradient of grassland degradation (α_SMA_ = 1.06, *p* = 0.17) (Figure 2), supporting allometric partitioning theory. However, with an increase in grassland degradation, the allometric relationship between AGB and BGB in NDG changed to an isometric relationship in degraded grasslands. The allometric relationships were 89%, 83%, 72%, and 68% of the scaling exponents of AGB versus BGB in LDG, MDG, HDG, and SDG, respectively. They differed significantly from 1.0 (*p* < 0.05) (supporting allometric partitioning theory) and were lower compared with that in NDG. In addition, BGB increased at a faster rate than AGB (Figure 2b).

We performed structural equation modeling (SEM) to understand how soil microbial richness influences plant biomass allocation and predicted the importance of these biotic variables based on a random forest model (Figure 2f). We found that AMF richness and diversity (Sobs, Chao1, and Shannon indices) were the most essential variables that strongly impacted the AGB/BGB ratio, while bacterial richness did not impact the BGB/AGB ratio. This finding agreed with the correlation analysis, suggesting a link between richer and more diverse microbial communities (Figure S2), particularly within the AMF and fungal taxa, and plant biomass allocation toward the root system. Therefore, our study revealed that the AMF in the plant roots exerted much stronger effects on plant biomass allocation patterns than soil bacteria and fungi.

### 3.4. Composition, Co-Occurrence Networks, and Keystone Taxa of AMF Communities

**Figure 3:**
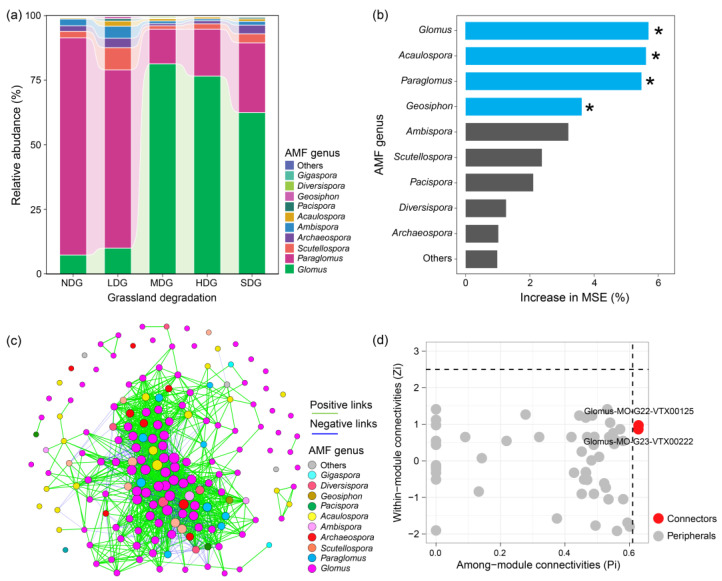
Community composition of AMF at the genus level in grasslands with different degrees of rocky desertification (**a**). Random forest modeling determined the importance of AMF genera in plant biomass allocation (**b**). * *p* < 0.05. Co-occurrence networks of the AMF community in grasslands with different degrees of rocky desertification (**c**). Identification of keystone taxa in the AMF community based on Zi and Pi (**d**). Zi is the z-score of the connectivity within the module of node i, while Pi is the participation coefficient P-scores of node i.

The AMF communities primarily consisted of members of the genera *Glomus*, *Paraglomus*, and *Scutellospora*, accounting for 93.47% of the relative abundance of all genera (Figure 3a). Relative to NDG and LDG, the abundance of *Glomus* was higher in MDG, HDG, and SDG. Areas with higher degradation showed a greater presence of *Glomus*, a type of beneficial soil fungus. On the other hand, the presence of another type of soil fungus, *Paraglomus*, decreased as the *Festuca ovina* grassland became more degraded. A random forest model was employed to pinpoint the AMF genera that exerted the most significant effect on the composition of the microbial community in *Festuca ovina* grasslands (Figure 3b). The significant importance of AMF genera in decreasing order was *Glomus* > *Acaulospora* > *Paraglomus* > *Geosiphon*. The analysis of species interactions within the fungal communities (co-occurrence network) revealed that several key genera emerged, such as *Glomus*, *Paraglomus*, *Scutellospora*, *Archaeospora*, and *Acaulospora* (Figure 3c). These key genera are likely important for maintaining the overall balance and functioning of the fungal community. To further investigate the roles of different AMF species within the network, we employed Zi–Pi plotting (Figure 3d). There were no nodes categorized as module hubs and network hubs, while only two nodes, VTX00125 and VTX00222 (belonging to the genus *Glomus*), could be allocated into network connectors. These keystone species, based on network analysis, are important for AMF communities and might exert a much stronger effect on plant biomass allocation patterns.

### 3.5. Effects of the Selected AMF Species Inoculation on Plant Biomass Allocation Under Rocky Desertification Stress

**Figure 4:**
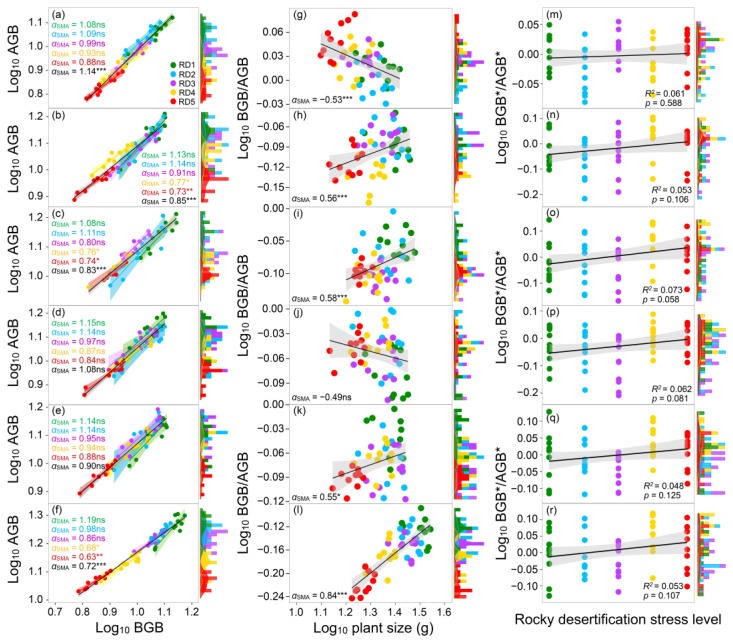
Standardized major axis (SMA) regressions between shoot and root biomasses of *Festuca ovina* inoculated without (**a**,**g**,**m**) or with *Glomus mosseae* (**b**,**h**,**n**), *Glomus intraradices* (**c**,**i**,**o**), *Acaulospora laevis* (**d**,**j**,**p**), *Diversispora spurca* (**e**,**k**,**q**), and four AMF species (**f**,**l**,**r**) under different rocky desertification stress conditions (RD1, RD2, RD3, RD4, and RD5). (**a**–**f**) General allometric relationships of shoot biomass (AGB) versus root biomass (BGB). (**g**–**i**) Standardized major axis (SMA) regressions between organ ratios and plant size of *Festuca ovina*. (**m**–**r**) Pearson correlation analysis between correlated BGB/AGB (BGB*/AGB*) ratio and rocky desertification stress level. The solid line and shaded areas show the best fit and 95% CI of the fit test, respectively. ns, not significant; * *p*  <  0.05; ** *p*  <  0.01; and *** *p* < 0.001.

AMF inoculation had significant effects on plant biomass allocation patterns under rocky desertification conditions (Figure 4). Rocky desertification stress promoted plant biomass allocation from shoot to root, while the *α*_SMA_ (0.88–1.08) of plants without AMF inoculation under each treatment did not differ from 1.0. On the contrary, *G. mosseae*, *R. intraradices*, and multiple AMF-colonized plants exhibited *α*_SMA_ lower than 1.0 (*p* < 0.05) under relatively high rocky desertification stress (RD4 and RD5). In addition, the BGB/AGB ratio of non-mycorrhizal plants showed a negative correlation with plant size (*p* < 0.001), while the BGB/AGB ratio of mycorrhizal plants had a positive correlation with plant size (*p* < 0.05). The biomass allocation pattern of non-mycorrhizal plants was inconsistent with optimal partitioning theory, as the BGB/AGB ratio showed no significant relationship with stress levels (*p* > 0.05).

### 3.6. Relationship Between Plant Nutrient Distribution and Plant Biomass Allocation

**Figure 5:**
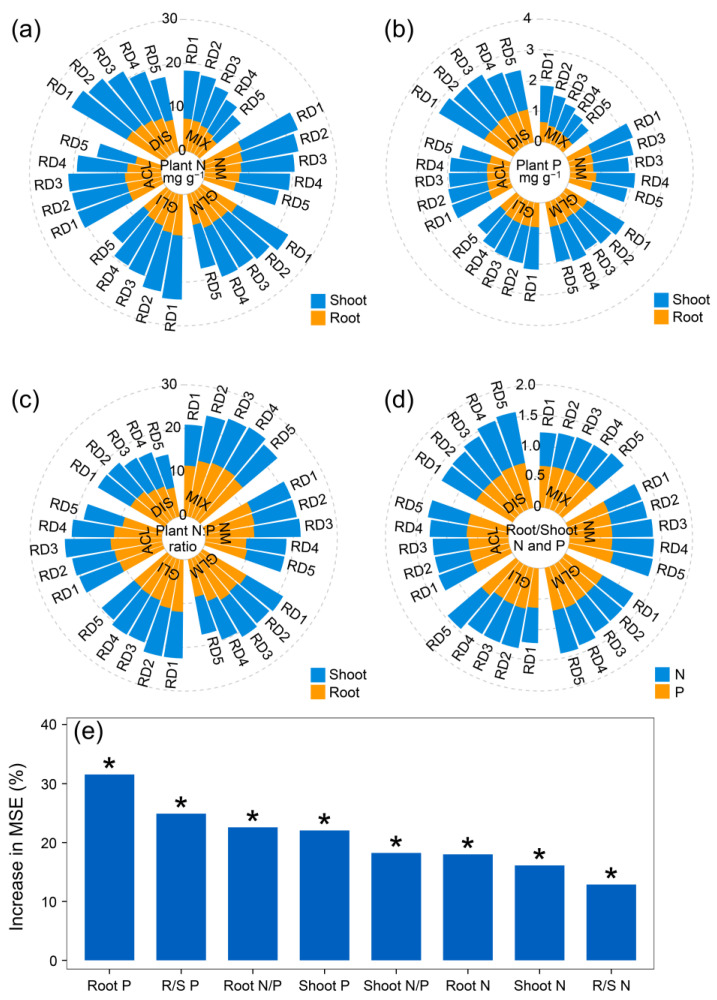
The N and P stoichiometry (shoot and root N (**a**), shoot and root P (**b**), shoot and root N:P ratio (**c**), and root/shoot N and P (**d**)) of *Festuca ovina* inoculated without (NM) or with *Glomus mosseae* (GLM), *Glomus intraradices* (GLI), *Acaulospora laevis* (ACL), *Diversispora spurca* (DIS), and mixture of four AMF species (MIX), and their contributions to plant biomass allocation patterns under different rocky desertification stress conditions (RD1, RD2, RD3, RD4, and RD5) based on random forest modeling (**e**). * *p* < 0.05.

Rocky desertification stress was shown to decrease the nitrogen (N) and phosphorus (P) in plant shoots and roots (Figure 5 and Figure S3). This is supported by the significant negative correlations observed. For instance, a strong negative correlation was observed between shoot N content and rocky desertification stress level (*r* = −0.559, *p* < 0.001). Negative trends were also noted for root N, shoot P, root P, and the N-to-P ratios in both shoots and roots. Interestingly, the proportion of nutrients allocated between roots and shoots responded differently to stress (Figure 5e and Figure S4). The ratio of root N to shoot N (R/S N) and the ratio of root P to shoot P (R/S P) increased with an increasing rocky desertification stress level (positive correlations with *r* = 0.171 and 0.455, respectively, and *p* < 0.001). This suggests that under rocky desertification stress, plants may prioritize allocating nutrients to their root systems, potentially to enhance water and nutrient uptake from soil.

## 4. Discussion

### 4.1. Plant Biomass Allocation Patterns in Festuca ovina Grasslands Along Rocky Desertification Gradient

Plant biomass allocation is considered the main process in structuring grassland ecosystems when responding to environmental changes [53]. Isometric partitioning theory or allometric partitioning theory predicts that the biomass partitioning between AGB and BGB is constant or inconstant [54], whereas optimal partitioning theory states that the ABG/BGB ratio will remain constant with a changing plant size, as environmental factors regulate the biomass allocation between AGB and BGB independent of plant size [55]. In this study, the overall trends in plant biomass allocation were consistent across the different degraded grasslands; there might be subtle variations specific to each site that our analysis missed. To examine this possibility, we further analyzed variation in the scaling slope under different degrees of grassland degradation. Our study suggested that the slopes did not present any difference from 1.0 in NDG ($\alpha_{SMA}\;$ = 0.91, *p* = 0.170). This finding supports previous studies [11,56] suggesting that the relationship between AGB and BGB tends to be proportional in grasslands and forests. This consistency suggests that the proportional allocation strategy might be common across various ecosystems. While previous studies found a consistent relationship between AGB and BGB across different environments, our findings indicated that the isometric pattern might not hold for plants growing in harsh conditions. For instance, the slopes between AGB versus BGB were significantly different from 1.0 in LDG ($\alpha_{SMA}\;$ = 0.89, *p* = 0.04), MDG ($\alpha_{SMA}\;$ = 0.83, *p* = 0.005), HDG ($\alpha_{SMA}\;$ = 0.72, *p* < 0.0017), and SDG ($\alpha_{SMA}\;$ = 0.68, *p* < 0.0017), revealing that *Festuca ovina* grassland degradation affects plant biomass allocation patterns.

Plants growing in degraded grasslands, where resources like water and nutrients are scarce, may prioritize allocating resources differently compared to plants in nutrient-rich environments. This could be an adaptive strategy to survive under harsh conditions. When essential nutrients are limited in degraded grasslands, plants might accumulate more carbohydrates (sugars) in their shoots. At the same time, they might invest more energy into growing their root systems to explore a larger area of soil rich in water and nutrients. This shift in resource allocation can result in a reduction in the proportion of plant biomass allocated to shoots (aboveground) compared to roots (belowground). This concept is consistent with previous findings that reported a similar trend in biomass allocation under nutrient-limited conditions [57]. In such scenarios, the allometric relationship between AGB and BGB (how these two parts scale with each other) is predicted to have a shallower slope, indicating a greater investment in the belowground organs. Moreover, soil rocky desertification seemed to be another factor influencing the plant biomass allocation of *Festuca ovina* grassland. The drought soils caused by grassland desertification can be toxic to plants, limit their water uptake, and create stress [58]. Plants with a well-developed root system can access water from deeper soil layers through root hairs. Stems provide support and transport water and nutrients, and leaves are the primary sites for photosynthesis, where plants capture sunlight to create energy. This creates a potential trade-off for plants in degraded grasslands. They may need to invest more resources in developing a larger root system to find water and nutrients in rocky-desertification-degraded soils. We found that an isometric relationship between AGB and BGB (constant allocation pattern) only existed in the natural grassland, which presumably had a lower salinity. In contrast, degraded grasslands with potentially higher salinity levels exhibited an allometric relationship. This suggests that plants in degraded grasslands might shift their biomass allocation patterns to prioritize root growth over shoot growth to cope with the harsh conditions.

However, when we analyzed all the plant samples together, the way that plants allocated resources between their aboveground parts (shoots) and belowground parts (roots) seemed to follow a consistent pattern. Grassland degradation enhanced biomass allocation to the belowground organs, leading to allometric growth. These results are consistent with a previous study which found that plants adapt to environmental changes by evolving their allometric patterns to maximize water and nutrient uptake under resource limitations [59]. Compared with aboveground competition for light, plants suffered from more belowground competition under nutrient-poor conditions (e.g., rocky desertification grassland).

### 4.2. AMF Had a Stronger Effect on PLANT Biomass Allocation than Bacteria and Fungi in the Grassland

Soil microbiota is crucial for plant health and growth, while the effects of microbial community composition and diversity on plant biomass allocation remain uncertain. In addition, bacteria and fungi are the dominant microorganisms in soils, but their relative importance to plant biomass allocation patterns is still unclear. In this study, we evaluated the different responses of the dominant microbial group to grassland degradation and found that, as compared to bacteria, fungi were more resistant to stress conditions, and the highest diversity was found in MDG (Figure 1). Interestingly, the diversity of AMF (belonging to fungal Glomeromycota) increased with an increase in the grassland degradation degree. The previous study indicated that AMF are widely distributed in soil and exhibit a high tolerance to ecosystem degradation [60]. This might be because AMF have developed several strategies to survive and even flourish in harsh environments, such as melanin, thick cell walls, and osmolytes [61].

Moreover, the current study revealed that *Festuca ovina* grassland degradation had an indirect effect on plant biomass allocation (BGB/AGB ratio) through influencing AMF diversity, which had a significant direct effect on the BGB/AGB ratio based on the structural equation model (Figure 2). Furthermore, AMF exhibited stronger effects on plant biomass allocation compared to bacteria and fungi according to the random forest model (Figure 2). These results can be attributed to the fact that AMF establish symbiotic associations with the roots of most terrestrial plants, enhancing their growth and productivity by facilitating the absorption of essential nutrients, including phosphorus, water, and minerals, particularly under stress conditions [62]. However, soil bacteria and fungi constitute a vast biological group, exhibiting a huge diversity of microbial taxa. They may form mutualistic relationships beneficial to plant growth (e.g., *Rhizobium*) or act as pathogens that inhibit plant growth (e.g., *Agrobacterium tumefaciens*). Ultimately, this results in no significant correlation between soil bacterial/fungal diversity and plant biomass allocation (Figure 2).

### 4.3. Mechanism of AMF on Plant Biomass Allocation in a Greenhouse Experiment

The biomass of *Festuca ovina* accounted for more than 90% of the total biomass in the studied health grassland. Therefore, in this study, we further selected *Festuca ovina* as the plant candidate to underpin the potential mechanism of AMF on plant biomass allocation patterns. According to the co-occurrence network analysis and random forest model, we identified that *Glomus*, *Paraglomus*, *Scutellospora*, *Acaulospora*, and *Geosiphon* contributed more to AMF communities than other genera (Figure 3), which were selected as the AMF inoculum. The results of the greenhouse experiment showed that the scaling slope for non-mycorrhizal plants was not different from 1.0, while that for multi-species inoculated plants was lower than 1.0 (Figure 4). Notably, the α_SMA_ observed in the multiple AMF treatments, relative to the single AMF treatment, was significantly reduced in harsh environments. This study indicated that introducing these helpful AMF to plant roots can influence how plants distribute their resources (aboveground vs. belowground) in different environments. Interestingly, mixed inoculation of these AMF species seemed to be more beneficial for root growth, especially when plants were facing harsh conditions. Our findings align with previous research, which showed that plants benefit more when exposed to a wider variety (from four to eight) of AMF strains from different families, compared to having just one type of AMF [63]. Functional complementation among different species, such as facilitation, could explain this higher efficiency, as already observed in this study. A meta-analysis also suggested that different types of these helpful fungi (AMF) have unique strengths [64]. For instance, some AMF strains (Glomeraceae) might be better at helping plants obtain nutrients and fight off fungal diseases, while others (Gigasporaceae) might be more effective at helping plants tolerate heavy metals in the soil. Additionally, it has already been demonstrated that different microbial taxa could have synergistic effects on plant performance, and the important role of microbial species interaction in plant health and ecosystem function has already been revealed [65,66], which might explain the significant effects of multi-species AMF inoculation on plant biomass allocation in this study.

Our study indicated that the effect of AMF on the content and stoichiometric balance of nutrient elements (especially P) may be one of the pathways affecting plant biomass allocation patterns. The N/P ratio in plant tissues has been widely used to estimate plant nutrient condition [67]. In our study, mycorrhizal plants had a higher P and lower root N/P ratio compared to non-mycorrhizal plants under rocky desertification stress conditions (Figure 5 and Figure S3). This was mainly because AMF have thread-like structures that extend far beyond the root zone, allowing them to explore a larger soil volume for P. Furthermore, the Growth Rate Hypothesis (GRH) suggests that faster-growing plants often exhibit higher concentrations of P and a lower N/P ratio, because rapidly growing plants need more P-rich RNA for protein synthesis [68]. Previous studies have shown that mycorrhizal plants have a high P content and invest more in RNA production [46]. Therefore, the reduced N/P ratio in mycorrhizal plants in our study might help plants to synthesize proteins more efficiently, leading to increased root growth and a greater investment of biomass in belowground tissues. Interestingly, plants inoculated with a mixture of four AMF strains showed the lowest N/P ratio under the highest rocky desertification stress levels (RD4 and RD5). The phenomenon suggests that the compatible mixture of AMF appeared to be more effective in regulating plant biomass allocation compared with a single AMF inoculation. This result is consistent with a previous study that showed that high AMF diversity has a greater potential to exert multiple functions than low AMF diversity due to the complementary functions existing among different AMF species [69]. The existence of high genetic variability among AMF strains is considered as one of the most important guarantees for the functional diversity of ecosystems, especially under stress conditions [70]. Alternatively, a mixture of AMF strains in plant roots can also promote opportunities for cooperation among different species to achieve complementation in the function of nutrient uptake and stress alleviation, which could be the mechanism through which high AMF diversity influences plant biomass trade-offs [71]. Our study revealed that AMF inoculation can be a major biotic driver influencing plant biomass allocation patterns. This conclusion is supported by the random forest model analysis and the observed effects of AMF on plant P uptake and root N/P ratio.

**Figure 6:**
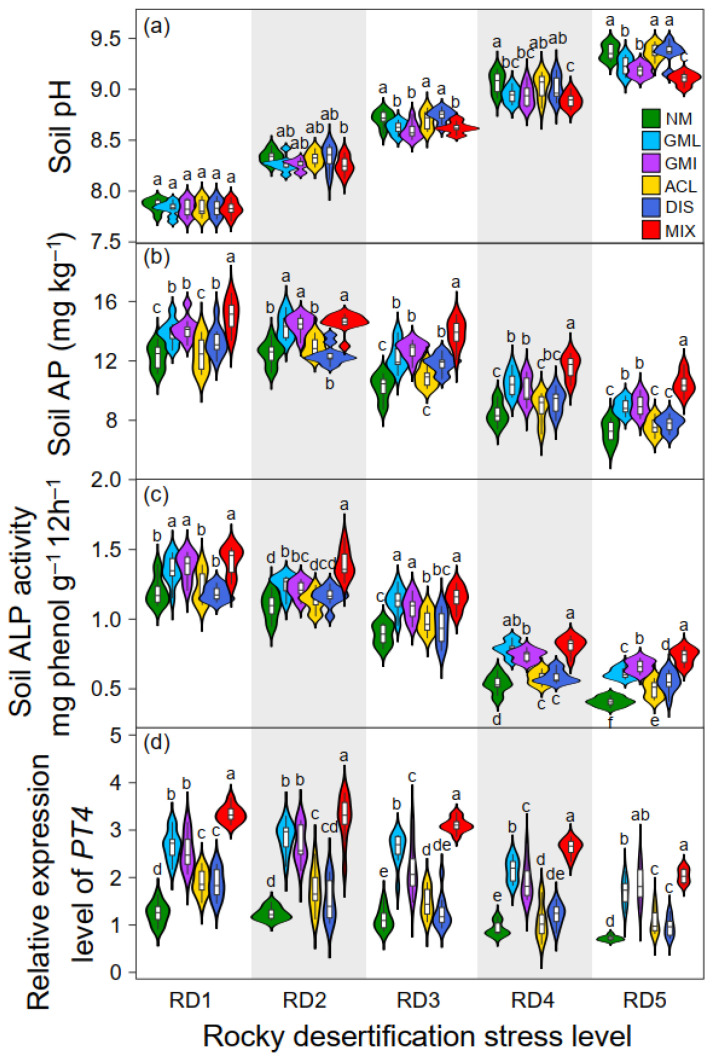
Soil pH (**a**), available phosphorus (AP) concentration (**b**), soil alkaline phosphatase (ALP) activity (**c**), and relative expression level of *PT4* gene (**d**) in the root of *Festuca ovina* without or with AMF inoculation under different rocky desertification stress conditions. Boxes or violins (means ± SD, *n* = 5) associated with the same letter for the same parameter do not differ significantly at *p* < 0.05 according to Duncan’s multiple range test.

We further assessed the soil pH, soil available P (AP), soil alkaline phosphatase (ALP) activity, and relative expression level of AMF-inducible phosphate transporters (*PT4*) in plant roots to underpin the mechanisms of AMF on plant biomass accumulation and allocation patterns. We found that *G. mosseae-*, *G. intraradices-*, and AMF-mixture-inoculated plants had a low soil pH, higher AP content, and ALP activity (Figure 6). P plays a critical role in sustaining plant growth and productivity. However, its availability in soils is frequently constrained by fixation mechanisms, often falling below the threshold required for optimal plant growth [72]. In contrast, the low-molecular-weight organic acids released by AMF can lower soil pH and mobilize P bound to Fe oxides [73]. Additionally, ALP is one of the main phosphatase enzymes involved in organic P mineralization. The inoculation of AMF can increase the activity of ALP, which breaks down organic P in the soil, making it more accessible to plants [74,75]. Combined with soil acidity because of pH reduction, AMF contribute to the high availability of P for plant absorption. Another strategy that AMF might employ to improve plant P uptake is influencing the expression of AMF-inducible genes (e.g., *PT4*) within the plant roots, as shown in a previous study [76]. By enhancing the expression level of the *PT* gene, AMF can help plants acquire more P from soils and promote biomass accumulation.

Generally, the plant roots that directly contact the soil are more vulnerable to soil degradation compared to aboveground parts. Our results indicated that mycorrhizal plants had a higher BGB/AGB ratio compared with non-mycorrhizal plants in the harsh environment (Figure 4). These results indicate that belowground growth strategies existed between the roots and shoots, which benefited the nutrient acquisition of plants for growth in rocky desertification stress conditions. The biomass allocation strategies of plants between BGB and AGB in stress conditions can be partly explained by their root nutrient absorption capacity. For example, during plant growth in soil with low levels of nutrients (especially P), more roots could increase the nutrient absorption area as a strategy to improve plant growth. Additionally, promoting the growth of plant roots could further increase the amount of carbohydrates secreted by plants into the soil, which can invest more C for AMF inoculation and indirectly improve nutrient acquisition through the mycorrhizal pathway [77]. Therefore, the current study is consistent with the hypothesis that a greater root biomass is beneficial for plant nutrient absorption, thereby maintaining a high plant growth rate in degraded grasslands or rocky desertification stress conditions [78].

This study advances the fundamental understanding of the plant biomass allocation mechanisms in karst grasslands with different degradation degrees. Our findings also hold timely relevance for grassland studies across varying environments. The key environmental drivers identified here, particularly the symbiotic fungi AMF, lay the groundwork for further exploration of ecosystem functioning and will inform future strategies to alleviate grassland degradation impacts on ecosystem structure, function, and stability. However, the observed variation patterns and driving factors of plant biomass allocation across grasslands relied on a limited number of investigations in this study, probably constrained by climate conditions, grassland types, and plant species. Therefore, future work should expand sampling to additional sites at large scales to strengthen the validation of these findings. Overall, multi-species AMF inoculation is strongly recommended as a practical intervention for restoring degraded karst grasslands. AMF enhance plant nutrient uptake, stress tolerance, and soil health, directly addressing key limitations in these harsh environments. However, large-scale application necessitates the careful consideration of several factors. Firstly, introducing multi-species AMF inoculants may disrupt indigenous AMF communities, with potential long-term ecological consequences. Secondly, the effectiveness of inoculation is highly context-dependent: outcomes can vary significantly based on local climate conditions, dominant vegetation types, and the specific plant species targeted for restoration. Finally, conducting thorough site-specific assessments before widespread implementation is crucial to evaluate initial effectiveness, potential risks, and optimize inoculant composition and application methods for the specific karst ecosystem.

## 5. Conclusions

Plant biomass allocation between AGB and BGB is one of the important strategies for plants’ adaptability to environmental changes. AMF were more effective in influencing plant biomass allocation (BGB/AGB ratio) compared to soil bacteria and fungi across different degradation degrees of *Festuca ovina* grassland. By using ecological network analysis and a random forest model, we selected AMF keystone species and revealed their effects on plant biomass allocation under rocky desertification stress conditions in a greenhouse experiment. The results showed that AMF inoculation was beneficial for plant biomass allocation to belowground parts mainly through reducing soil pH and increasing the activity of ALP and expression level of *PT4*, thereby promoting the bioavailability of P in the soil, P content, and N/P ratio in plant roots. Overall, this study highlights the crucial role of AMF in plant growth and adaptation to stressful environments via regulating plant biomass allocation patterns. Further study is still required to examine the change patterns of other macronutrients (i.e., potassium, calcium, and magnesium) and micronutrients (i.e., iron, copper, and zinc) and their effects on plant biomass allocation in karst ecosystems to obtain more universal conclusions.

## Figures and Tables

**Table 1:** Soil properties in grasslands with different degrees of degradation.

Soil Properties	Study Sites				
	NDG	LDG	MDG	HDG	SDG
pH	6.39 ± 0.59 c	6.66 ± 0.48 c	7.45 ± 0.80 b	7.82 ± 0.58 a	7.97 ± 0.56 a
EC (μS m^−1^)	270 ± 25.8 c	368 ± 23.3 b	398 ± 21.5 b	439 ± 17.5 a	475 ± 58.6 a
CEC (cmol kg^−1^)	0.35 ± 0.02 a	0.35 ± 0.02 a	0.36 ± 0.04 a	0.35 ± 0.03 a	0.38 ± 0.03 a
ESP (%)	2.81 ± 0.50 c	3.08 ± 0.28 bc	2.49 ± 0.36 ab	3.40 ± 0.18 a	3.07 ± 0.31 ab
SAR (cmol^0.5^ kg^−0.5^)	1.90 ± 0.15 c	2.16 ± 0.08 bc	2.22 ± 0.25 b	2.33 ± 0.11 b	2.71 ± 0.14 a
SOC (mg g^−1^)	56.9 ± 9.57 ab	57.8 ± 8.73 ab	60.1 ± 9.76 a	53.5 ± 8.19 b	43.5 ± 8.06 b
TP (mg g^−1^)	0.46 ± 0.02 a	0.44 ± 0.02 ab	0.39 ± 0.01 c	0.41 ± 0.03 bc	0.42 ± 0.03 bc
AP (mg kg^−1^)	7.53 ± 0.85 a	7.44 ± 0.63ab	6.82 ± 0.63 b	5.58 ± 0.53 c	4.85 ± 0.24 d
TN (mg g^−1^)	2.00 ± 0.11 a	2.01 ± 0.13 a	1.84 ± 0.07 a	1.89 ± 0.08 a	1.92 ± 0.15 a
AN (mg kg^−1^)	2.63 ± 0.26 a	2.56 ± 0.17 a	2.53 ± 0.11 ab	2.28 ± 0.20 bc	2.27 ± 0.21 c

The values (means ± SD, *n* = 5) followed by the same letter in each row do not differ significantly at *p* < 0.05 according to Duncan’s multiple range test. NDG, non-degraded grassland; LDG, lightly degraded grassland; MDG, moderately degraded grassland; HDG, heavily degraded grassland; SDG, severely degraded grassland; EC, electrical conductivity; CEC, cation exchange capacity; ESP, exchangeable sodium percentage; SAR, sodium adsorption ratio; TOC, total organic carbon; TP, total phosphorus; AP, available phosphorus; TN, total nitrogen; AN, available nitrogen.

## Data Availability

The original contributions presented in this study are included in the article/Supplementary Material. Further inquiries can be directed to the corresponding author.

## Supplementary Materials

The following supporting information can be downloaded at: https://www.mdpi.com/article/10.3390/jof11070525/s1, Table S1: Descriptions of vegetation coverage and plant aboveground biomass, soil pH, and electrical conductivity (EC) for grasslands of five degraded degrees in this study; Table S2: DNA sequences of PCR primers used in quantitative real−time PCR (qRT−PCR) determination of phosphate transporters (*PT4*) gene copy number in roots of *Festuca ovina* under different treatments; Figure S1: Location map of *Festuca ovina* grasslands in the karst area, Guizhou province, China.; Figure S2: Relationships between BGB/AGB ratio, richness indices (Sobs, Chao1), and diversity index (Shannon) of bacterial (a–c), fungal (d–f), and AMF (g–i) communities; Figure S3: Relationships between shoot N (a), root N (b), shoot P (c), root P (d), shoot N/P ratio (e), root N/P ratio (f), R/S N (g), R/S P (h), and rocky desertification stress levels; Figure S4: Relationships between shoot N, root N, shoot P, root P, shoot N/P ratio, root N/P ratio, R/S N, R/S P, and BGB/AGB ratio.

## Abbreviations

The following abbreviations are used in this manuscript:

ACL	*Acaulospora laevis*
AGB	Aboveground biomass
ALP	Alkaline phosphatase
AMF	Arbuscular mycorrhizal fungi
AN	Available nitrogen
AP	Available phosphorus
BGB	Belowground biomass
CEC	Cation exchange capacity
DIS	*Diversispora spurca*
EC	Electrical conductivity
ESP	Exchangeable sodium percentage
GLI	*Glomus intraradices*
GLM	*Glomus mosseae*
HDG	Heavily degraded grassland
LDG	Lightly degraded grassland
MDG	Moderately degraded grassland
MIX	Mixture of four AMF species
NDG	Non-degraded grassland
NM	Inoculated without AMF
*PT*	Phosphate transporter
RD	Rocky desertification
SAR	Sodium adsorption ratio
SDG	Severely degraded grassland
SEM	Structural equation model
SMA	Standardized major axis
SOC	Soil organic carbon
TN	Total nitrogen
TP	Total phosphorus
