# Supplementary material for: Communities of Arbuscular Mycorrhizal Fungi and Their Effects on Plant Biomass Allocation Patterns in Degraded Karst Grasslands of Southwest China
Source: J Fungi (Basel). 2025 Jul 16;11(7):525. doi: 10.3390/jof11070525 (PMC12300158; doi:10.3390/jof11070525)
Supplement: Supplementary file 1 [file jof-11-00525-s001.zip › jof-3731828-supplementary.pdf]

Supplementary Materials

Communities of Arbuscular Mycorrhizal Fungi and Their Effects on Plant Biomass Allocation Patterns in Degraded Karst Grasslands of Southwest China

Wangjun Li [1], Xiaolong Bai [1], Dongpeng Lv [2], and Yurong Yang [3,*]

1 College of Ecological Engineering, Guizhou University of Engineering Science, Bijie 551700, China; teesn470@gues.edu.cn (W.L.); baixiaolong@gues.edu.cn (X.B.)

2 Key Laboratory of Ecological Microbial Remediation Technology of Yunnan Higher Education Institutes, Dali University, Dali 671003, China; 911882@dali.edu.cn

3 State Environmental Protection Key Laboratory of Wetland Ecology and Vegetation Restoration, School of Environment, Northeast Normal University, Changchun 130117, China

* Correspondence: yangyr422@nenu.edu.cn

Supplementary Materials and Methods

Experiment 1: field investigation

DNA extraction, PCR amplification, and Illumina sequencing

For the analysis of bacterial and fungal communities, total DNA was extracted using approximately 0.5 grams of frozen soil sample according to the DNA extraction kit manufacturer's instructions (OMEGA, USA). After extracting DNA, the diameter of soil DNA was evaluated through 1% agarose gel electrophoresis, and a NanoDrop 2000 nucleic acid quantifier (Thermo Scientific Inc., Waltham, MA, USA) was employed to ascertain the concentration and purity of the DNA extracted. For the bacterial community, the V3-V4 regions of the 16S rRNA gene were targeted for amplification with the 338F/806R universal primers [36-38], while the ITS gene were amplified using ITS1/ITS2 universal primers for the fungal community [39].

To investigate the AMF communities, we employed a commercially available soil DNA extraction kit (MOBIO, Carlsbad, USA) to isolate total DNA from root samples of *Festuca ovina*. We then utilized nested PCR, a two-step amplification process, to target and amplify specific regions of fungal ribosomal RNA (rRNA) genes. The first PCR round employed a primer pair (AML1/AML2) specific to Glomeromycota fungi [40], resulting in amplified DNA fragments of approximately 800 base pairs (bp) in length. Following this, a more specific primer set (AMV4.5NF/AMDGR) was utilized to further amplify a smaller (~300 bp) target region within the fungal 18S rRNA gene [41]. By employing this nested PCR approach, we achieved a high degree of specificity and sensitivity in detecting and characterizing the AMF communities associated with the plant roots. The PCR amplifications were carried out in 20 μL mixtures with the following protocol based on our previous study [42]. Once amplified, the DNA fragments (amplicons) were quality-checked and pooled together in equal amounts. This pooled sample was then subjected to a paired-end sequencing process on an Illumina MiSeq platform (Majorbio, Shanghai, China). Paired-end sequencing essentially involves reading both ends of each DNA fragment, generating high-quality sequencing data (2 × 300 bp). To ensure transparency and data reproducibility, the raw sequencing data obtained in this study have been deposited in a public database: the National Center for Biotechnology Information Sequence Read Archive (NCBI SRA) under the accession numbers of PRJNA1277251, PRJNA1277264, and PRJNA674743.

The raw sequencing reads of the 16S rRNA, ITS, SSU genes underwent a series of processing steps to ensure data quality. Briefly, the reads were demultiplexed and subjected to quality filtering using fastp version 0.20.0. Subsequently, paired-end reads were merged using FLASH version 1.2.7. Operational Taxonomic Units (OTUs) were allocated to the processed sequences using UPARSE version 7.1, employing a 97% similarity threshold, and excluding any chimeric sequences identified by Uchime. For taxonomic analysis, the Ribosomal Database Project (RDP) Classifier algorithm, SILVA (version 119), UNITE (version 7.0), and MaarjAM databases were

employed, with a confidence threshold of 70% applied. Before further analysis, resampling was conducted to account for variations in sequence numbers across all soil samples. These rigorous processing steps ensured the reliability and accuracy of the microbial community data. The complexity of AMF diversity at different study sites was reflected by the index of observed species (Sobs), abundance-based coverage estimator (ACE), Chao1, and Shannon, which were calculated in R software (version 3.6.2) using the vegan package [43].

Experiment 2: greenhouse experiment

Growth substrate, plant seeds, and fungal inoculum

Soil samples were collected from the top layer (0–20 cm, non-degraded grassland) in the same karst area. The obtained soil samples were air-dried for 20 days and then passed through a 2-mm sieve to remove root-stone residue and ensure homogeneity. The sieved soils used for growth substrate have the following properties: pH 6.4 (1: 5, soil: water, m/v); 57.3 mg g^{-1} of soil organic matter; 0.47 mg g^{-1} of total phosphorus; 7.43 mg kg^{-1} of available phosphorus; 2.18 mg g^{-1} of total nitrogen; 2.15 mg kg^{-1} of available nitrogen; and 131.6 mg kg^{-1} of available potassium; 279 μS cm^{-1} of electrical conductivity. The sieved soil was then placed into a clean cloth bag and autoclaved at 121°C for 1.5 h two times to eliminate all possible mycorrhizal propagules and other soil microorganisms, which was used as the growth substrate in this study.

The *Festuca ovina* seeds, which were obtained from the same field station in July, were surface sterilized with sodium hypochlorite (0.5%, v/v) for 5 min, washed three times with sterile water, treated with 70% ethanol for 5 min, and then washed three times again with sterile water. Surface sterilized seeds were pre-germinated on moist filter paper Petri dishes (9 cm in diameter) in the dark for 2 days at 28°C before they were transferred into plastic pots.

Four AMF strains, *Glomus mosseae* (GLM, now *Funneliformi mosseae*), *Glomus intraradices* (GLI, now *Rhizophagus intraradices*), *Acaulospora laevis* (ACL), and *Diversispora spurca* (DIS) were were isolated onsite. The fungus was propagated on *Festuca ovina* and white clover (*Trifolium repens* L.) grown in pot cultures with high soil salinity for 6 months to adapt rocky desertification stress. The AMF inoculum consisted of a mixture of spores, mycelium, colonized root fragments, and air-dried substrate.

Experimental design

The experiment was conducted in the greenhouse of Guizhou University of Engineering Science, Bijie, China for 95 days. The temperature ranged from 20-35°C and the relative air humidity ranged from 55-87%. The experiment consisted of a randomized complete block design with six inoculation treatments: (1) control plants did not inoculate with AMF (NM), (2) plants inoculated with AMF *Glomus mosseae* strain (GLM), (3) plants inoculated with AMF *Glomus intraradices* strain (GLI), (4) plants inoculated with AMF *Acaulospora laevis* strain (ACL), (5) plants

inoculated with AMF *Diversispora spurca* strain (DIS), (6) plants inoculated with a mixture of the five AMF strains (MIX). Five degrees of grassland rocky desertification were created through regulating the variation of soil-to-gravel ratio [45]): RD1, 100% soil; RD2, a mixture of 85% soil and 15% gravel; RD3, a mixture of 70% soil and 30% gravel; RD4, a mixture of 55% soil and 45% gravel; RD5, a mixture of 40% soil and 60% gravel. Each treatment had five replicates for a total of 150 plastic pots.

The pre-germinated seeds of *Festuca ovina* were transplanted into each plastic pot (20 cm upper diameter, 15 cm lower diameter, and 15 cm depth) filled with growth substrate. In each pot of AMF inoculation treatment, AMF inoculum was placed 2 cm below the seeds and covered with substrate. Non-mycorrhizal control plants received 15 mL of the filtered leachate (20 μm) from AMF inoculum and sterilized AMF inoculum to correct possible differences in microbial communities and root biomass. After emergence, seedlings were thinned to a final density of 4 plants per pot. During the first 10 days, seedlings were grown without the addition of neutral and alkaline salts to obtain plants with functional mycorrhizas and avoid stress effects on AMF symbiosis establishment. A saucer was placed under each pot to retain water and other nutrients. The plants were watered every 2 days and each pot was irrigated with 50 mL of half-strength Hoagland's nutrient solution every month throughout the experiment.

References

36. Huse, S.M., Dethlefsen, L., Huber, J.A. Exploring microbial diversity and taxonomy using SSU rRNA hypervariable tag sequencing. *PLoS Genet*. **2008**, *4*, e1000255.
37. Caporaso, J.G., Lauber, C.L., Walters, W.A., Berg-Lyons, D., Lozupone, C.A., Turnbaugh, P.J., Fierer, N., Knight, R. Global patterns of 16S rRNA diversity at a depth of millions of sequences per sample. *Proc. Natl. Acad. Sci. U.S.A.* **2011**, *108*, 4516–4522.
38. Salas-González, I., Reyt, G., Flis, P., Custódio, V., Gopaulchan, D., Bakhoum, N., Dew, T.P., Suresh, K., Franke, R.B., Dangl, J.L., Salt, D.E. Coordination between microbiota and root endodermis supports plant mineral nutrient homeostasis. *Science* **2021**, *371*, eabd0695.
39. Schoch, C.L., Seifert, K.A., Huhndorf, S., Robert, V., Spouge, J.L., Levesque, C.A., Chen, W., Fungal Barcoding Consortium, Fungal Barcoding Consortium Author List, Bolchacova, E., et al. Nuclear ribosomal internal transcribed spacer (ITS) region as a universal DNA barcode marker for Fungi. *Proc. Natl. Acad. Sci. U.S.A.* **2012**, *109*, 6241–6246.
40. Lee, J., Lee, S., Young, J.P.W. Improved PCR primers for the detection and identification of arbuscular mycorrhizal fungi. *FEMS Microbiol. Ecol*. **2008**, *65*, 339–349.
41. Lumini, E., Orgiazzi, A., Borriello, R., Bonfante, P., Bianciotto, V. Disclosing arbuscular mycorrhizal fungal biodiversity in soil through a land-use gradient using a pyrosequencing approach. *Environ. Microbiol.* **2010**, *12*, 2165–2179.
42. Ban, Y., Jiang, Y., Li, M., Zhang, X., Zhang, S., Wu, Y., Xu, Z. Homogenous stands of a wetland grass living in heavy metal polluted wetlands harbor diverse consortia of arbuscular mycorrhizal fungi. *Chemosphere* **2017**, *181*, 699–709.
43. Oksanen, J., Kindt, R., Legendre, P., O'Hara, B., Stevens, M.H., Oksanen, M.J., Suggests, M.A. The vegan package. *Community Ecol.* 2007, *10*, 719
45. Shen, K.; He, Y.; Xu, X.; Umer, M.; Liu, X.; Xia, T.; Guo, Y.; Wu, B.; Xu, H.; Zang, L.; et al. Effects of AMF on plant nutrition and growth depend on substrate gravel content and patchiness in the karst species *Bidens pilosa* L. *Front. Plant Sci.* **2022**, *13*, 968719.

Supplemental tables

Table S1. Descriptions of vegetation coverage and plant aboveground biomass, soil pH, and electrical conductivity (EC) for grasslands of five degraded degrees in this study.

Degraded degree	Vegetation coverage (%)	Aboveground biomass (g m^{-2})	pH	Electrical conductivity (μs cm^{-1})
NDG	80−100	300−400	6.0−6.5	200−300
LDG	65−85	250−350	6.5−7.0	300−350
MDG	45−70	200−300	7.0−7.5	300−400
HDG	30−50	150−250	7.0−8.0	350−450
SDG	<30	100−200	7.5−8.5	400−500

NDG, non-degraded grassland; LDG, lightly degraded grassland; MDG, moderately degraded grassland; HDG, heavily degraded grassland; SDG, severely degraded grassland.

Table S2. DNA sequences of PCR primers used in quantitative real−time PCR (qRT−PCR) determination of phosphate transporters (*PT4*) gene copy number in roots of *Festuca ovina* under different treatments.

Gene	Primer sequence (5′−3′)	T_m (°C)
β-actin	F: GCCAACAGAGAGAAGATGACC	58.37
	R: ATAGAGGGAAAGCACCGCCT	60.98
PT4	F: TTCATCGCAGCCGTCTTTG	58.84
	R: GGCATCTTCATCCGCCAGTAG	60.88

Supplemental figures.

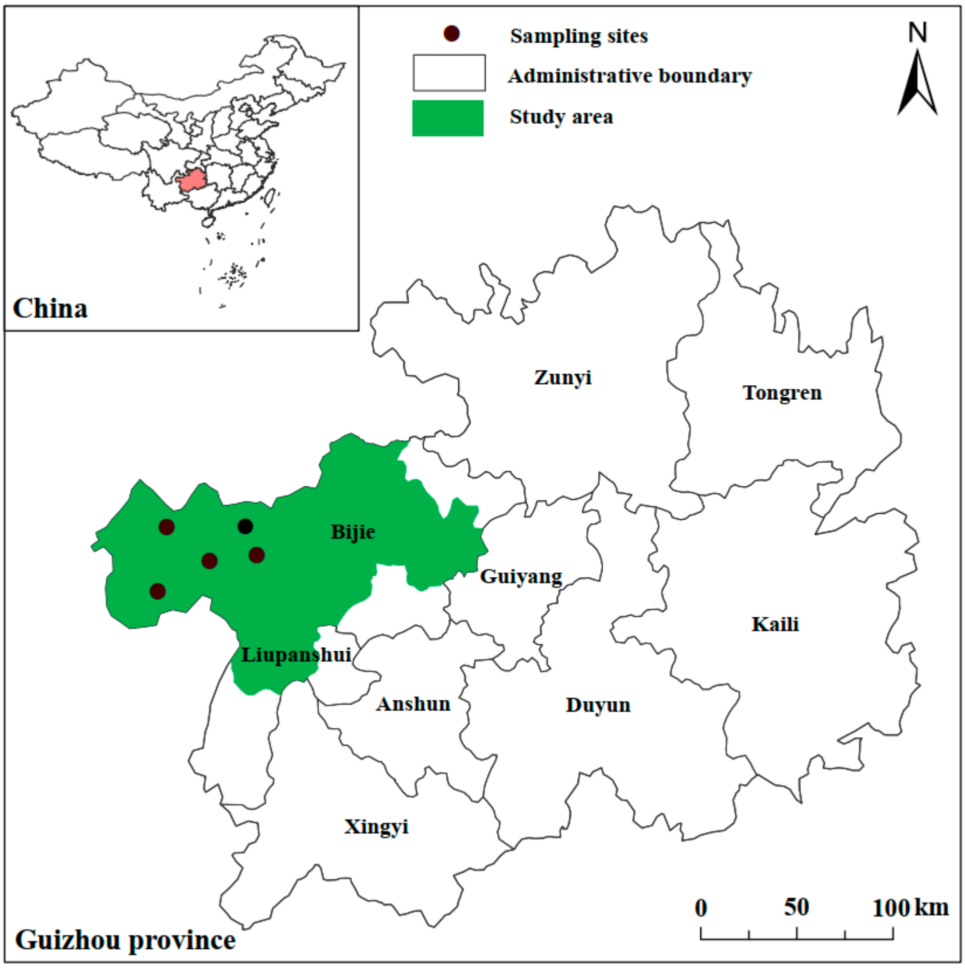

Figure S1. Location map of the karst area showing *Festuca ovina* grasslands in Bijie city, Guizhou province, China.

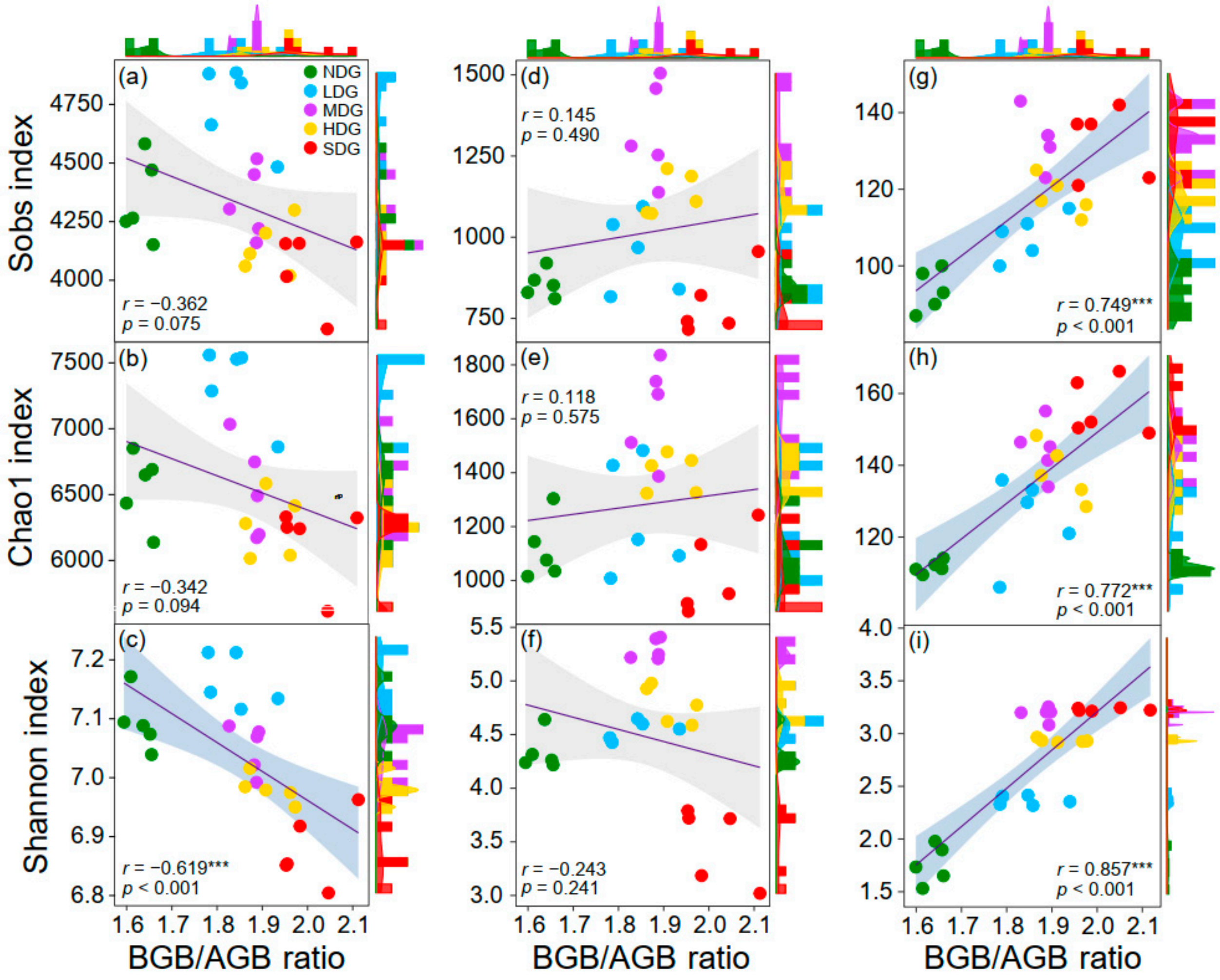

Figure S2. Relationships between BGB/AGB ratio, richness indices (Sobs, Chao1), and diversity index (Shannon) of bacterial (a, b, and c), fungal (d, e, and f), and AMF (g, h, and i) communities. NDG, non-degraded grassland; LDG, lightly degraded grassland; MDG, moderately degraded grassland; HDG, heavily degraded grassland; SDG, severely degraded grassland light gray and blue bands represent 95% confidence intervals. ***$p < 0.01$.

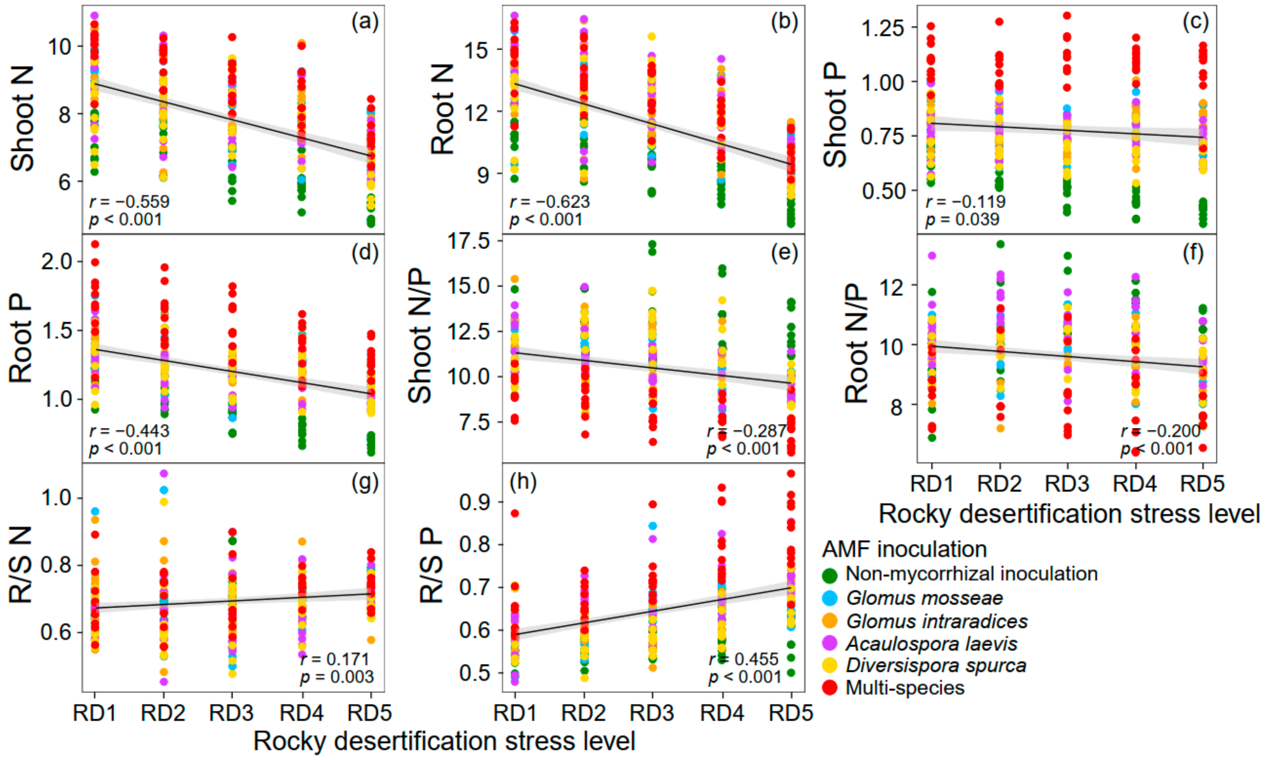

Figure S3. Relationships between shoot N (a), root N (b), shoot P (c), root P (d), shoot N/P ratio (e), root N/P ratio (f), R/S N (g), R/S P (h) and rocky desertification stress levels. Light gray and black bands represent 95% confidence intervals.

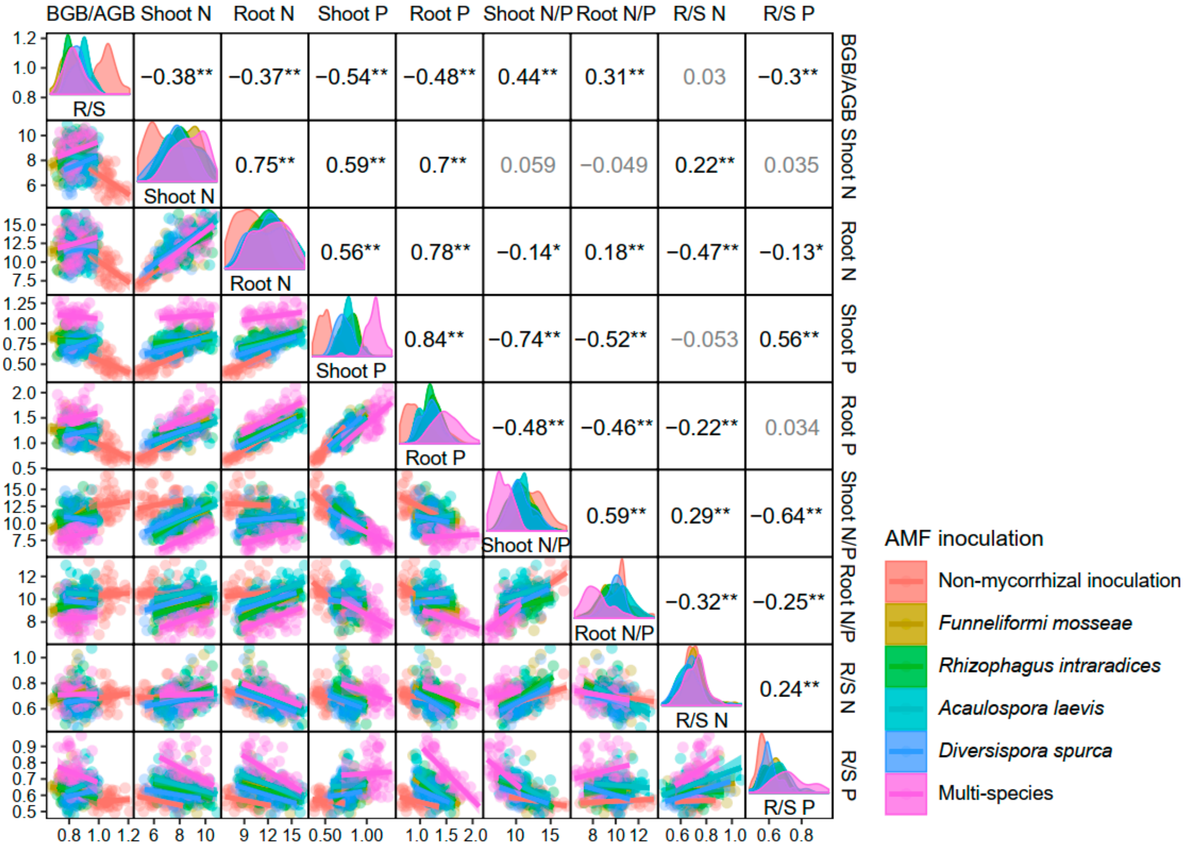

Figure S4. Relationships between shoot N, root N, shoot P, root P, shoot N/P ratio, root N/P ratio, R/S N, R/S P, and BGB/AGB ratio. ***$p < 0.01$; and *$p < 0.05$.
